# Successful management of severe IRIS associated with disseminated histoplasmosis in an HIV patient using cytokine adsorption therapy and JAK inhibition

**DOI:** 10.1186/s12981-025-00819-4

**Published:** 2025-11-19

**Authors:** Micha Banz, Benjamin Schleenvoigt, Niklas Eckardt, Michael Baier, Dunja Wilmes, Diana Dudziak, Mathias W. Pletz

**Affiliations:** 1 Institute of Infectious Diseases and Infection Control, Jena University Hospital, Jena, Germany; 2 Department of Internal Medicine IV (Gastroenterology, Hepatology, and Infectious Diseases), Jena University Hospital, Jena, Germany; 3 Institute of Diagnostic and Interventional Radiology, Jena University Hospital, Jena, Germany; 4 Institute of Medical Microbiology and Hygiene, Jena University Hospital, Jena, Germany; 5 Robert Koch Institute, Berlin, Germany; 6 Institute of Immunology, Jena University Hospital, Jena, Germany

**Keywords:** Immune reconstitution inflammatory syndrome, Histoplasmosis, HIV/AIDS, Opportunistic infections, JAK inhibitor, Cytokine adsorption therapy

## Abstract

We report on the successful management of severe immune reconstitution inflammatory syndrome (IRIS) in a 28-year-old Indonesian male with advanced HIV/AIDS, complicated by disseminated histoplasmosis. This case highlights the clinical challenges and innovative approaches in treating severe IRIS, where conventional management strategies proved inadequate. The patient presented with progressive clinical deterioration briefly after initiation of antiretroviral therapy (ART). Disseminated histoplasmosis was suspected based on clinical and radiographic findings and later confirmed as the underlying infectious trigger of IRIS, guiding targeted therapeutic strategies. Clinical management involved the novel use of a Janus kinase (JAK) inhibitor and a cytokine adsorption filter (CytoSorb^®^), alongside escalated antifungal and immunosuppressive therapies. This multifaceted approach not only stabilized the patient’s condition but also highlighted the importance of considering advanced therapeutic options in severe IRIS cases. The use of JAK inhibition in this context provides new insights into the modulation of immune responses in IRIS, while cytokine adsorption therapy offered a method to control the systemic inflammatory response that characterizes this condition. This case underscores the critical need for awareness of uncommon pathogens like Histoplasma in IRIS and illustrates the potential for integrating novel therapeutic modalities to improve outcomes in these complex scenarios. Our experience suggests that early consideration of advanced immunomodulatory therapies should be considered in severe IRIS cases refractory to standard treatments.

## Background

Immune reconstitution inflammatory syndrome (IRIS) is either characterized by a paradoxical worsening of a patient’s clinical condition following the initiation of anti-infective therapy which is called *paradoxical IRIS* or by the flare-up of an existing, previously undiagnosed infection, the *unmasking IRIS*. This phenomenon arises from the recovering immune system mounting an exaggerated inflammatory response against opportunistic pathogens and was initially reported in HIV patients receiving antiretroviral therapy (ART). While IRIS is commonly associated with HIV and infections such as tuberculosis and cryptococcosis, it can also be precipitated by rare fungal infections like histoplasmosis, which can lead to severe and potentially fatal episodes of IRIS [1]. The severity of IRIS often provides insights into the underlying pathogen, with *Histoplasma capsulatum* and *Cryptococcus neoformans* frequently linked to life-threatening cases [1]. In the case presented, disseminated histoplasmosis was suspected early as the underlying trigger of IRIS in an immunosuppressed HIV patient and was later confirmed through molecular diagnostics. The patient suffered severe IRIS with systemic hyperinflammation and multiorgan dysfunction, which was effectively managed through escalated immunosuppression, including the use of a JAK inhibitor, alongside the implementation of a cytokine adsorption filter. To our knowledge, this is the first documented and published case of tailored immunomodulatory treatment using cytokine adsorption and JAK inhibition to manage a devastating case of IRIS.

## Case Presetation

On November 25th 2022, a 28-year-old homosexual male from Indonesia, who had been living in Germany for over four years, was diagnosed with advanced HIV/AIDS during outpatient evaluation for symptoms of dyspnea and the microbiological confirmation of *Pneumocystis jirovecii* pneumonia (PCP) and esophageal candidiasis. Laboratory results revealed profound immunosuppression with a CD4 + T-cell count: 4 cells/µL; CD4/CD8 ratio: 0.89; HIV viral load: 2,010,000 copies/mL, consistent with CDC Stage C3 disease [2]. He was started on high-dose intravenous trimethoprim-sulfamethoxazole for PCP, fluconazole for esophageal candidiasis, and after two more weeks started on antiretroviral therapy (ART) with a fixed-dose combination of bictegravir, emtricitabine, and tenofovir alafenamide.

**Fig. 1:**
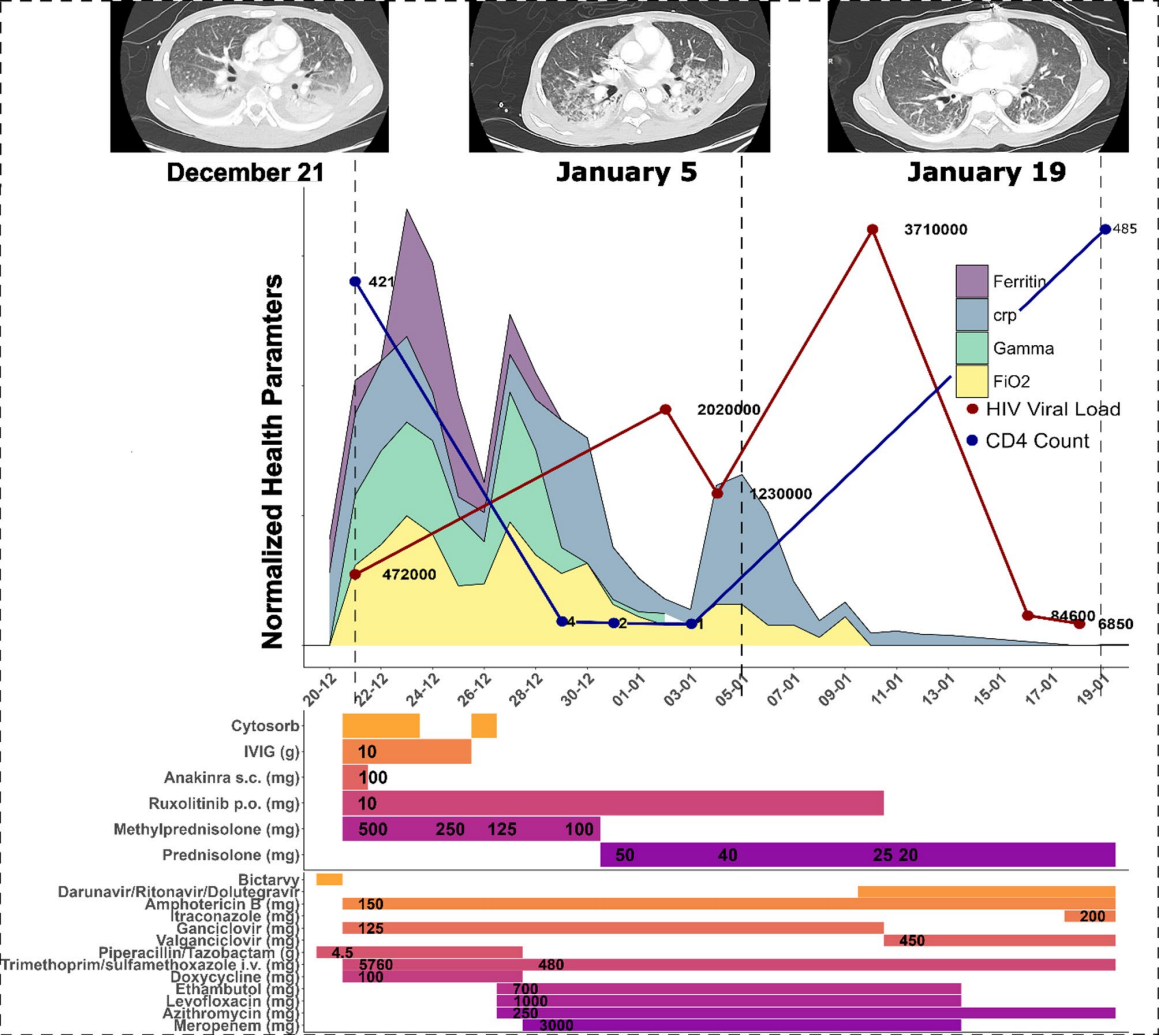
Clinical and Therapeutic Timeline: The figure illustrates the patient’s clinical course and treatment from December 21, 2022, to January 19, 2023. The main panel shows density plots with normalized trajectories of key inflammatory and clinical parameters, including Ferritin, C-reactive protein (CRP), norepinephrine requirement (Gamma), and the fraction of inspired oxygen (FiO2​). Overlaid on this graph are the specific data points for the patient’s HIV viral load (red dots) and CD4 + T-cell count (blue dots), with their corresponding values labeled. The middle panel details the timeline and dosages of administered immunosuppressive therapies, including cytokine adsorption (CytoSorb^®^), intravenous immunoglobulins (IVIG), Anakinra, Ruxolitinib, Methylprednisolone, and Prednisolone. The bottom panel presents the timeline and dosages of anti-infective and antiretroviral therapies

On December 21st, 2022, two weeks after initiating ART, the patient was transferred to our hospital due to rapid clinical deterioration. Laboratory findings at that time revealed a paradoxical response: despite a profound immunological recovery with his CD4 + T-cell count increasing from 4 to 421 cells/µL, his HIV viral load had not suppressed and was measured at 472,000 copies/mL (Fig. 1). This was accompanied by worsening symptoms including persistent fever, weight loss, and abdominal discomfort, which led to his transfer to the intensive care unit (ICU) for progressive dyspnea and acute respiratory failure. At ICU admission, he showed severe wasting (BMI: 15.9), pancytopenia, and required mechanical ventilation.

The combination of rapid immune recovery under ART, followed by a rapid clinical decline and a decreasing CD4 + T-cell count, strongly suggested the development of severe immune reconstitution inflammatory syndrome (IRIS). Considering the life-threatening respiratory failure and critical condition, the team made the decision to pause ART to mitigate further immune activation in the context of life-threatening hyperinflammation. While discontinuation of ART is generally not recommended, current guidelines allow for interruption in exceptional cases where corticosteroids fail to achieve improvement [3].

Given the patient’s origin from an endemic region, the miliary radiological pattern, and systemic deterioration despite ongoing treatment with high-dose trimethoprim-sulfamethoxazole and fluconazole, disseminated histoplasmosis was considered early as a likely underlying cause of IRIS and empiric antifungal therapy was initiated.

Bronchoalveolar lavage fluid (BALF) obtained on admission revealed *Pneumocystis jirovecii*, cytomegalovirus (CMV), and *Pseudomonas aeruginosa*. In response, antimicrobial therapy was escalated to include piperacillin/tazobactam at 4.5 g every six hours, doxycycline at 100 mg twice daily, empiric amphotericin B at 0.7 mg/kg daily for the suspected IRIS-trigger and ganciclovir at 5 mg/kg twice daily. Although histopathologic confirmation of CMV pneumonitis was not available, PCR positivity in BALF, the patient’s critical immunosuppression, and the anticipated use of further immunosuppressive agents led to the precautionary initiation of antiviral therapy. Methylprednisolone was administered at 500 mg/day for three days but failed to improve respiratory or hemodynamic status.

The patient remained critically ill, requiring high inspired oxygen fractions (FiO₂ up to 0.84) and vasopressor support. Laboratory and imaging findings at that time fulfilled with five out of eight of the HLH-2004 criteria [4]: persistent fever, hepatosplenomegaly, pancytopenia, hypofibrinogenemia of 0.9 g/L (< 1.5 g/L) and markedly elevated ferritin concentrations of 138,740 µg/L (>100,000 µg/L).

Due to steroid-refractory IRIS/HLH and progressive clinical deterioration, immunosuppressive therapy was escalated. The patient received intravenous immunoglobulin (IVIG, 400 mg/kg/day), the interleukin-1 receptor antagonist anakinra (100 mg daily), the Janus kinase (JAK) inhibitor ruxolitinib (5 mg twice daily for a total of 16 days), and cytokine adsorption therapy using a CytoSorb^®^ filter integrated into a continuous renal replacement therapy (CRRT) circuit for a duration of five days [5].

Bone marrow aspirates ruled out mycobacterial and leishmanial infections and revealed no signs of hemophagocytes. Given the clinical and radiographic overlap with tuberculosis and the patient’s origin from an endemic region, disseminated histoplasmosis was considered [6]. The diagnosis of disseminated histoplasmosis was robustly confirmed on December 31, 2022, through a multi-modal approach performed by the German National Reference Center for Histoplasmosis at the Robert Koch Institute. This included both a positive antigen test and a positive PCR from serum and BALF, providing strong evidence from different methodologies. The diagnosis was further solidified by an additional positive 18 S-PCR from a bone marrow sample, confirming systemic involvement [7].

The patient gradually improved under combined antifungal treatment (liposomal amphotericin B until January 19, 2023, followed by itraconazole) and intensified immunosuppression. The patient’s co-infections with Pneumocystis jirovecii, CMV, and Pseudomonas aeruginosa were successfully managed, as evidenced by a decline in inflammatory markers like C-reactive protein, negative follow-up blood cultures, and sustained clinical improvement. Due to persisting anemia, ganciclovir switched to prophylactic dose after 21 days of therapeutic dosing with a subsequent increase in hemoglobin. However, briefly afterwards, the patient reported bilateral vision loss and ophthalmological examination revealed suspected bilateral CMV retinitis, which was supported by rising number of CMV copies in blood. CMV-retinitis was successfully managed with oral valganciclovir – a re-decrease of hemoglobin was to be tolerated – and laser treatment. On January 10, 2023, ART was reinitiated using a protease-inhibitor–based regimen comprising darunavir, ritonavir, and dolutegravir. Viral suppression and immune recovery were evident, with the CD4 + T-cell count rising to 485 cells/µL by January 19th. Respiratory function improved steadily, follow-up imaging demonstrated resolution of pulmonary infiltrates, and the patient was discharged in stable condition, eventually returning to work.

## Discussion

A critical point of discussion is the diagnosis of IRIS, as the patient’s HIV viral load had not declined by the time of transfer to our facility. However, the data from the two weeks prior show a profound immunological response to ART, with a rapid CD4 + T-cell increase from a baseline of 4 to 421 cells/µL. This explosive immune recovery, immediately preceding a life-threatening hyperinflammatory deterioration, is the hallmark of an unmasking IRIS. This constellation suggests that the immunological reconstitution was sufficient to trigger IRIS, even before virological suppression was achieved, a phenomenon that can occur in cases of severe initial immunosuppression, potentially compounded by factors like incipient malabsorption in a critically ill patient. This case emphasizes the critical need to consider rare opportunistic infections such as histoplasmosis in patients experiencing severe IRIS, particularly those from endemic regions. *Histoplasma capsulatum* can clinically and radiographically resemble tuberculosis, making it an essential differential diagnosis in these contexts. The severity of IRIS often correlates with specific pathogens; in particular, life-threatening IRIS is frequently associated with infections caused by *Histoplasma capsulatum* and *Cryptococcus neoformans* [8, 9]. In our patient, the rapid clinical deterioration shortly after ART initiation, combined with a miliary radiological pattern and the patient’s geographic background, prompted early empirical antifungal therapy. The subsequent confirmation of histoplasmosis via PCR from bone marrow validated this approach and supports a high index of suspicion in similar presentations.

Management of this life-threatening case required a multifaceted immunomodulatory strategy after standard corticosteroid therapy failed. The patient received intravenous immunoglobulin, the JAK-inhibitor ruxolitinib, and extracorporeal cytokine adsorption via use of CytoSorb^®^ filter. The CytoSorb^®^ filter, which received its CE certification in 2011, is a hemoadsorption device designed to remove a broad spectrum of inflammatory cytokines from the blood. This reduction can buy the time for immunosuppressive therapies to take effect, facilitating hemodynamic stabilization and improved organ function. The use of JAK inhibitors, such as ruxolitinib—first approved by the FDA in 2011 for myelofibrosis—is particularly relevant in this scenario. These agents modulate the signaling pathways of various cytokines involved in the inflammatory cascade by inhibiting the Janus kinase family of enzymes (JAK1/2), offering a broader immunosuppressive effect than therapies targeting single cytokines. JAK inhibitors such as ruxolitinib modulate cytokine signaling across multiple inflammatory pathways and have shown efficacy in other hyperinflammatory conditions [10–12]. CytoSorb^®^ therapy is designed to adsorb a wide range of circulating pro-inflammatory cytokines [13–16], and in our case, it provided hemodynamic stabilization and a therapeutic window for immunosuppressive therapy to take effect [17], despite a brief inflammatory flare after therapy cessation.

While previous case reports have documented the efficacy of interleukin inhibitors such as anakinra and tocilizumab in managing severe IRIS [18–20]. The use of JAK inhibitors like ruxolitinib represents a more novel therapeutic strategy in this context. An advantage of JAK inhibitors in contrast to many other immunosuppressants is that their effect is not long lasting after discontinuation. The combination of antifungal treatment, immunosuppressive escalation, including both interleukin blockade and JAK inhibition, and cytokine adsorption was instrumental in stabilizing the patient’s very critical condition. Such an aggressive immunomodulatory strategy carries the significant risk of secondary infections, particularly in a patient with severe AIDS, and its use was carefully considered only due to the life-threatening, steroid-refractory nature of the hyperinflammation. Recent reports further support the potential of JAK inhibition in AIDS-related hyperinflammation, including Talaromyces-associated HLH [21] and paradoxical tuberculosis-IRIS [22]. This case illustrates the potential benefit of integrating targeted immunomodulatory approaches in the management of complex IRIS presentations triggered by uncommon opportunistic infections [23].

## Conclusion

Clinicians should maintain a high index of suspicion for rare opportunistic infections in patients presenting with severe IRIS, particularly when standard treatments fail and the patient originates from an endemic area. Histoplasmosis should be considered a differential diagnosis in such contexts, given its ability to mimic tuberculosis. The severity of IRIS can provide insights into the underlying pathogen and guide therapeutic strategies.

The combination of escalated immunosuppression, including novel agents like JAK-inhibitors, and cytokine adsorption therapy using a CytoSorb^®^ filter may represent a promising intervention for life-threatening IRIS. Although the CytoSorb^®^ filter does not address the underlying cause of inflammation, its ability to temporarily reduce cytokine levels can create a therapeutic window for immunosuppressive agents to take effect. Further research is warranted to evaluate the efficacy and safety of these interventions in broader patient populations.

## Data Availability

All data relevant to this case report are included within the manuscript. Additional information can be obtained from the corresponding author upon reasonable request.

## Abbreviations

HIV: Human Immunodeficiency Virus
AIDS: Acquired Immunodeficiency Syndrome
IRIS: Immune Reconstitution Inflammatory Syndrome
ART: Antiretroviral Therapy
PCP: Pneumocystis jirovecii Pneumonia
CMV: Cytomegalovirus
FiO₂: Fraction of Inspired Oxygen
HLH: Hemophagocytic Lymphohistiocytosis
IVIG: Intravenous Immunoglobulin
JAK: Janus Kinase
ICU: Intensive Care Unit
BMI: Body Mass Index
BALF: Bronchoalveolar Lavage Fluid
CDC: Centers for Disease Control and Prevention
DNA: Deoxyribonucleic Acid
mg: Milligram
µg: Microgram
g: Gram
kg: Kilogram
min: Minute
bw: Body Weight
CRP: C-Reactive Protein
